# The Impact of Food Waste Compost, Vermicompost, and Chemical Fertilizers on the Growth Measurement of Red Radish (*Raphanus sativus*): A Sustainability Perspective in the United Arab Emirates

**DOI:** 10.3390/foods13111608

**Published:** 2024-05-22

**Authors:** Sara B. Almaramah, Abdelghafar M. Abu-Elsaoud, Wejdan A. Alteneiji, Shaikha T. Albedwawi, Khaled A. El-Tarabily, Seham M. Al Raish

**Affiliations:** 1 Department of Biology, College of Science, United Arab Emirates University, Al Ain 15551, United Arab Emirates; 201808015@uaeu.ac.ae (S.B.A.); 201900372@uaeu.ac.ae (W.A.A.); 201900644@uaeu.ac.ae (S.T.A.); ktarabily@uaeu.ac.ae (K.A.E.-T.); 2 Department of Botany and Microbiology, Faculty of Science, Suez Canal University, Ismailia 41522, Egypt; amsmohamed@imamu.edu.sa; 3 Department of Biology, College of Science, Imam Mohammad Ibn Saud Islamic University (IMSIU), Riyadh 11623, Saudi Arabia

**Keywords:** crop yield, environmental sustainability, organic farming, plant growth promotion, sustainable agriculture

## Abstract

The pressing need for sustainable agricultural practices, especially with the increasing population, has directed attention towards alternative fertilizers that enhance crop yield while preserving soil integrity and reducing food loss. The current study investigated the comparative efficacy of food waste compost (FOWC), vermicompost, and chemical fertilizers on the growth of red radish. The present work used a systematic experimental design to evaluate plant growth parameters, including radish weight and height. The soil quality was determined by measuring the pH and electrical conductivity for all soil samples. The results indicated a significant variation in red radish fresh weight among different treatments. For example, the 25% vegetable and fruit waste compost (VFWC) treatment demonstrated a relatively high mean fresh weight, while the 50% mixed compost (MC) treatment yielded a much lower mean fresh weight. These numbers underscore the potential efficacy of specific food waste treatments in enhancing plant growth, with vermicompost at 50% and VFWC at 25% showing considerable promise in increasing crop yield. The current study concluded that FOWC and vermicompost significantly improved plant growth, advocating for their use as sustainable and environmentally friendly alternatives to chemical fertilizers. The current findings emphasized the importance of selecting appropriate fertilizer types and concentrations to optimize agricultural productivity and environmental sustainability, supporting the incorporation of food waste into agricultural systems as a beneficial resource.

## 1. Introduction

The rapid and continuous urban population growth is leading to a simultaneous rise in food demands and food waste [1]. This situation leads to multiple issues, such as waste management, limited agricultural resources, and decreased soil fertility [1,2]. The lack of soil fertility requires a continuous supply of fertilizers and nutrients [2]. The population’s continuous growth also leads to rapid food waste, and the unregulated anaerobic breakdown of waste at specific landfill sites leads to methane and carbon dioxide emissions, exacerbating global warming [3]. The wastes also emit toxic gases and foul odors, pollute groundwater through leachate, and decrease landfill space [3,4,5,6,7].

Kumar et al. [8] reported that 42% of food waste comes from households, 39% from food industries, and 5% occurs during distribution [8]. To achieve development goals for a sustainable environment, reducing food waste is an essential component [9]. Food waste negatively affects the environment and the agriculture industry [9]. The United Arab Emirates (UAE) spends around USD 4 billion annually (equivalent to AED 14.69 billion), making up around 40% of the daily domestic waste in the UAE [7,10].

Implementing sustainable management practices for food waste has become a significant obstacle [11], necessitating the development of novel approaches that alleviate environmental issues and improve agricultural output [11]. A practical and positive strategy involves repurposing diverse food waste materials, such as vegetables, fruits, meat, and bread waste, as organic compost [12]. This approach aligns with the principles of the circular economy and ecological sustainability, simultaneously offering a comprehensive solution to tackle waste management and agricultural productivity [11,12]. A recent study revealed the advantages of using food waste as a valuable resource in the farming industry [12].

Manure is the term used for fertilizer derived from plant and animal waste that positively impacts soil’s physical, chemical, and biological characteristics, as well as the soil’s fauna and nutrient content [3,13,14]. Organic residue comprises a variety of vital nutrients, including nitrogen, phosphorus, calcium, sulfur, magnesium, potassium, iron, and zinc [14]. These nutrients are crucial for achieving high crop yields, reducing the need for chemical fertilizers, and enhancing soil properties [15]. Nevertheless, the incorrect application of chemical fertilizers can result in adverse environmental consequences, including soil deterioration, water contamination, and the release of greenhouse gases [15,16].

Inorganic fertilizers are employed to enhance the productivity of crops and vegetables and augment the soil’s water-holding capacity [17,18]. There is a rising trend to reduce the frequency of applying inorganic fertilizers to soils by improving the efficiency of soil nutrient utilization and increasing the utilization of organic matter, and this could be achieved by using vermicompost and food waste compost (FOWC) [17]. Vermicompost is also widely acknowledged as having significant potential as a soil amendment among various sources of organic matter [18]. Many farmers utilize vermicompost to maximize their benefits while minimizing negative environmental effects [18].

Using FOWC and vermicompost is a sustainable and environmentally conscious approach to enhance agricultural productivity [18,19]. Instead of allowing food waste to contribute to environmental degradation and landfills, its conversion into nutrient-rich compost provides a valuable resource for augmenting soil fertility [18]. By transforming food waste into compost, scientists can decrease the impact of waste and establish a loop system that promotes the circular economy [19]. This innovative approach deals with waste management issues and agricultural productivity and fosters a more sustainable food production system [19,20]. These practices align with the global goal of promoting responsible and eco-friendly farming methods [18,19,20,21].

Research has demonstrated that adding vermicompost to soils can enhance the sprouting, development, and productivity of various vegetables, ornamental plants, and crops, such as cowpeas, cress, grapes, Chinese cabbage, bananas, strawberries, and tomatoes [22,23,24].

One way to apply food waste to soil is by composting it [22]. Composting is highly effective and cost-efficient. However, there are certain obstacles to overcome, such as the probability of odor emissions occurring during the biodegradation process and the extensive duration of the processing times [24,25]. Various methods have been proposed to decrease the time it takes to process household compost [26]. One such technique involves using a thermophilic composter in the shape of a drum, which can be adjusted to different temperatures [27]. The second method is composting using mechanical methods. This technique allows for the activation of microbial metabolism and increases microbiological activity [7,13,25,26,27,28,29].

Both of these techniques have the potential to generate a soil amendment that is rich in nutrients and can be utilized to enhance the growth of garden beds, lawns, and houseplants [27,28]. Additionally, these machines aid in curbing pollution caused by methane emissions and safeguarding essential topsoil [28,29]. Composting technologies encompass a range of methods, including aerobic composting, vermicomposting, anaerobic digestion, and in-vessel composting [13,28,29].

Red radish (*Raphanus sativus* L.) is a perennial root vegetable crop classified under the Brassicaceae family. The red radish’s raw or sprouted seeds, leaves, and roots can be consumed independently or as part of a salad [30]. The radish root epidermis exhibits a range of hues, including white, red, pink, purple, and yellow [30]. However, the root’s flesh is uniformly white and possesses a sharp, crunchy taste. The root skin exhibits a crimson hue due to anthocyanin pigment [30]. The root contains ample vitamins, glucosinolates, sulforaphane, polyphenolic compounds, sulfur, calcium, potassium, and phosphorus. Vegetables from the Brassicaceae family are linked to notable health advantages because they contain biologically active and powerful antioxidant compounds [30].

Managing food waste, producing sufficient food, and implementing sustainable agriculture are all areas that need to be addressed to accommodate our growing population, particularly in desert countries with limited resources [31,32,33]. Consequently, the current research highlights the significance of utilizing food waste, including that of vegetables, fruits, meat, rice, and bread, as organic fertilizers in two hypotheses: an earthworm-based method and a thermophilic machine.

In addition, the present study evaluated the potential effects of FOWC, compared to vermicompost and chemical fertilizers, on the production of radishes and soil pH and electrical conductivity (EC). The present investigation also provides a comprehensive understanding of the benefits of incorporating FOWC into agricultural systems, which can help modern farmers implement informed and environmentally conscious practices in contemporary farming.

## 2. Materials and Methods

### 2.1. Experimental Site and Design

The current study was conducted in the greenhouse at the Biology Department, College of Science, United Arab Emirates University (GPS 24.200250579134575, 55.67575985048151) during the winter seasons (January to April) of 2023.

The experiment was set in a split–split plot design, with FOWC, vermicompost, chemical fertilizer, and control (only seed starter potting mix) randomly distributed within the sub-plots. Each experimental sub-plot consisted of eight rows, with three replicates in random order. In each pot, there were two seeds (Table 1).

Our goal was to examine the effects of FOWC, vermicompost, and chemical fertilizer on the soil pH, EC, and growth characteristics of organic red radish seeds (*R. sativus*). In these experiments, the FOWC, vermicompost, chemical fertilizer, and control treatments used were prepared as follows.

#### 2.1.1. Control (C)

Seed starter potting mix, manufactured by Gardener’s in the United Arab Emirates, was used as the control treatment with the following product specifications: the basic material (dry soil): decomposed plant material, density: >200 kg/m^3^, organic matter: 88%, moisture content: 47%, EC) <1.5 millimhos per centimeter (mmhos/cm), salt content: <1.5 g/L, and pH of 5.5–6.5. The optimal EC range for growing radish is 1.0–1.5 ms/cm (1000–1500 μs/cm) [34].

#### 2.1.2. Food Waste Compost (FOWC)

The food waste was obtained from the preparation area of the students’ canteen located within the kitchen and from volunteering staff and students from the UAE university. It consisted of inedible raw food items.

The collection period lasted from January to February 2023. The food waste included vegetables, fruits, meat, chicken, fish, white-cooked rice, and pasta. Bread leftovers and scraps were cleansed with water, diced into small pieces, and promptly deposited into the composting device within 48 h of collection. Throughout this period, the food waste was stored in a plastic bag within the laboratory cabinet, maintaining a temperature of 21 °C.

The FOWC was made of food waste mixed with used paper cups and used papers and processed using an electric compost bin kitchen (Cavdle WasteCycler DCEC01, Guangdong, China), which composts the food waste at 120 °C for 2–4 h, depending on the waste volume under pressure. The cylindrical 3 L composter has dimensions of 25.3 cm × 25.3 cm × 31.5 cm, and weighs 7.12 kg.

##### Vegetable Waste Compost (VWC)

VWC: This was a mixture of any leftover raw vegetables and scraps used in different percentages and mixed with the seed starter potting mix from the first day: (1) 10% VWC = 10 parts vegetable waste, and 90 parts seed starter potting mix, (2) 25% VWC = 25 parts vegetable wastes, and 75 parts seed starter potting mix, and (3) 50% VWC = 50 parts vegetable waste, and 50 parts seed starter potting mix.

##### Fruit Waste Compost (FRWC)

FRWC: From the first day, a mixture of fruit leftovers and scraps was used in different percentages with seed starter potting mix: (1) 10% FRWC = 10 parts of fruit waste, and 90 parts seed starter potting mix, (2) 25% FRWC = 25 parts of fruit waste, and 75 parts seed starter potting mix, and (3) 50% FRWC = 50 parts of fruit waste, and 50 parts seed starter potting mix.

##### Vegetable and Fruit Waste Compost (VFWC)

VFWC: This was made from a mixture of any fruit and vegetable leftovers and scraps with the same amount of both of them, mixed in different percentages with seed starter potting mix from the first day as follows: (1) 10% VFWC = 10 parts of VFWC, and 90 parts seed starter potting mix, (2) 25% VFWC = 25 parts of VFWC, and 75 parts seed starter potting mix, and (3) 50% VFWC = 50 parts of VFWC, and 50 parts seed starter potting mix.

##### Meat, Fish, and Chicken Waste Compost (MFCWC)

MFCWC was collected from the canteen preparation area, and the sources were as follows, (1) meat: sheep or cow meat, flesh and bones were used, (2) fish: different fish types, bones, skins, and heads were used, and (3) chicken: bones, skin, and little flesh were used. MFCWC were mixed in different percentages with seed starter potting mix from the first day as follows: (1) 10% MFCWC = 10 parts of meat, fish, and chicken waste, and 90 parts seed starter potting mix, (2) 25% MFCWC = 25 parts of meat, fish, and chicken waste, and 75 parts GM, and (3) 50% MFCWC 50% = 50 parts of meat, fish, and chicken waste, and 50 parts GM.

##### Bread, Pasta, and Rice Waste Compost (BPRC)

BPRC %: Bread of different types, pasta, and rice were each cooked with boiling water, and a little salt was mixed in different percentages with seed starter potting mix from the first day as follows: (1) 10% BPRC = 10 parts bread of different types, pasta, and rice waste, and 90 parts seed starter potting mix, (2) 25% BPRC = 25 parts bread of different types, pasta, and rice waste, and 75 parts seed starter potting mix, and (3) 50% BPRC = 50 parts bread of different types, pasta, and rice waste, and 50 parts seed starter potting mix.

##### Mixed Compost (MC)

MC: This was made from a mixture of green materials, mainly from food waste and brown materials, such as leaves, stems, waste desk papers, and cup papers, with a carbon: nitrogen ratio of 30:1 [35]. This was mixed in different percentages with the seed starter potting mix from the first day as follows: (1) 10% MC = 10 parts green and brown materials, and 90 parts seed starter potting mix, (2) 25% MC = 25 parts green and brown materials, and 75 parts seed starter potting mix, and (3) 50% MC 50% = 50 parts of green and brown materials, and 50 parts seed starter potting mix.

#### 2.1.3. Vermicompost (V)

V: Vermicomposting is a natural process whereby earthworms (*Eisenia fetida*, known as red wigglers) convert waste material with rigid structures into compost. Mixed food waste was used to feed earthworms. Then, the vermicompost products were mixed with the GM in different percentages from the first day as follows: (1) 10% V = 10 parts of vermicompost product, and 90 parts seed starter potting mix, (2) 25% V = 25 parts of vermicompost product, and 75 parts seed starter potting mix, and (3) 50% V = 50 parts of vermicompost product, and 50 parts seed starter potting mix.

#### 2.1.4. Chemical Fertilizer (CF)

One gram of chemical fertilizer was mixed with 1000 mL of water to create a solution with 1.2 EC. It was applied in the second and third weeks after planting the seeds. The powder comprised 20% N, 20% P_2_O_5_, and 20% K_2_O + microelements. It was applied by fertigation (administering fertilizer solutions alongside irrigation water, usually via a micro-sprinkler or drip system) [36].

### 2.2. Greenhouse Experiments

Table 1 displays the treatments used in the current study, including the control group, as well as all the composts and chemical fertilizers. Control and amended soils were maintained in the greenhouse (15 h day/9 h night; temperature of 28 ± 2 °C; relative humidity of 42 ± 5%).

All sub-plots received all the FOWC from day one, while chemical fertilizer was added after two weeks. The red radish (*R. sativus*) was grown in January 2023. All plots received the same amount of water (150 mL) on alternate days. 

### 2.3. Plant Growth Measurements

The plant parameters measurements included the following.

#### 2.3.1. Radish Height

1. Height of the fresh radishes (including shoots, leaves, and roots) was measured using a tape measure by unit (cm).
2. Height of the shoot of the fresh radish was measured using a tape measure by unit (cm).
3. Height of the root of the fresh radish was measured using a tape measure by unit (cm).
4. Taproot top perimeter: perimeter of the middle fresh radish was measured using a tape measure in the units (cm).

#### 2.3.2. Radish Weight

The weight of the whole fresh and dry radish was measured using an analytical weighing scale.

#### 2.3.3. Leaf Surface Area (LSA)

LSA was measured by the grid count method, which uses the leaf’s dimensions (width and length). The measurement unit was in cm^2^ [37,38].

### 2.4. Determination of Total Bacterial Population in Soil Samples

Using a nutrient agar medium, the bacteria were isolated from each soil sample using the soil dilution plate method [39]. The nutrient agar (Lab M Limited, Lancashire, United Kingdom) was amended with cycloheximide and nystatin (50 µg mL^−1^ each; Sigma-Aldrich Chemie GmbH, Taufkirchen, Germany). These two antibiotics were amended with the nutrient agar just before pouring the plates. In Erlenmeyer flasks, 10 g of each soil was combined with 100 mL of sterile deionized water. The soil suspension was then agitated for 30 min at 28 °C using a rotary shaker (Model G76, New Brunswick Scientific, Edison, NJ, USA) set to 250 revolutions per minute (rpm).

After shaking the flasks, 0.2 mL aliquots were dispersed using a sterile hockey stick-shaped glass rod over the surface of nutrient agar in Petri plates after being diluted tenfold (10^−2^–10^−5^) [39] with sterile deionized water. The total bacterial population in each of the soils was determined [39].

### 2.5. Effect of Different Treatments on Soil pH, and EC

Soil quality was determined by measuring the soil pH and EC. The pH and EC were measured using an HEM conductivity meter, Technical Jica (Japan Cooperation, Tokyo, Japan) [40,41].

### 2.6. Statistical Analyses

The statistical package for social science (SPSS 29; SAS Institute Inc., Cary, NC, USA) was used for data analysis. Initial data analysis involved computing each treatment group’s basic descriptive statistics (mean and standard deviation), which provided a preliminary understanding of the data distribution and the central tendency. All data were subjected to analysis of variance (ANOVA). ANOVA was conducted to compare the effects of different fertilizers on each growth parameter. This test helped identify significant differences between the mean values of various groups. Mean values of treatments were compared using Tukey’s HSD test at *p* = 0.05.

## 3. Results

Plant length, biomass, LSA, soil pH and EC, and *R. sativus* parameters were measured as critical indicators of plant growth in this extensive study that evaluated the effects of different treatments on the growth of red radish plants. These treatments included various types of food waste, vermicompost, and chemical fertilizers.

**Figure 1:**
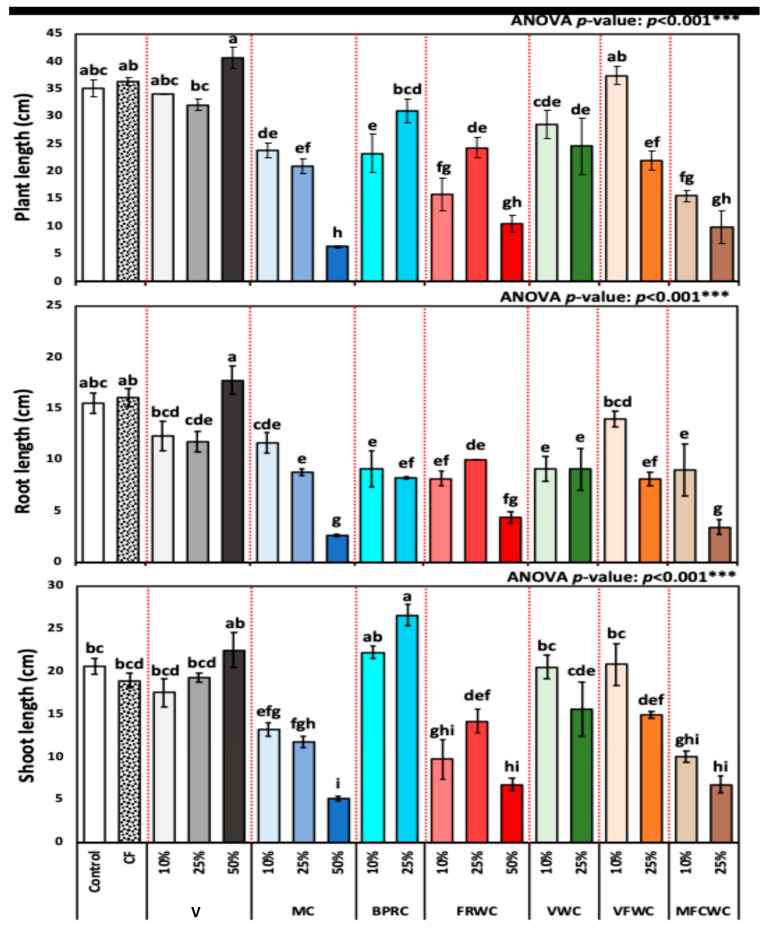
Plant length (cm), root length (cm), and shoot length (cm) of *R. sativus* treated with different treatments, including chemical fertilizer (CF), vermicompost (V), mixed compost (MC), bread, pasta, and rice waste compost (BPRC), fruit waste compost (FRWC), vegetable waste compost (VWC), vegetable and fruit waste compost (VFWC), and meat, fish, and chicken waste compost (MFCWC). Bars followed by different letters are significantly different according to Tukey’s HSD at 0.05 level. ***: Highly significant at *p* < 0.001, as revealed by one-way ANOVA. The red dotted line differentiates between different treatments.

Figure 1 shows the plant length, root length, and shoot length (cm) of *R. sativus* treated with different treatments, including chemical fertilizer, vermicompost (10, 25, and 50%), MC (10, 25, and 50%), BPRC (10, 25, and 50%), FRWC (10, 25, and 50%), VWC (10, and 25%), VFWC (10, and 25%), and MFCWC (10, and 25%).

The *R. sativus* plant length for different treatment groups from control, chemical fertilizers, vermicompost (10, 25, and 50%), VFWC (10 and 25%), BPRC (10 and 25%), MC (10, 25, and 50%), FRWC (10, 25, and 50%), MFCWC (10 and 25%), and VWC (10 and 25%) was 35.2 ± 1.53, 36.3 ± 0.73, 34.0 ± 0.00, 32.1 ± 0.97, 40.6 ± 1.93, 23.8 ± 1.39, 21.0 ± 1.29, 6.3 ± 0.25, 23.3 ± 3.52, 31.0 ± 2.10, 15.8 ± 2.95, 24.3 ± 1.77, 10.5 ± 1.59, 28.5 ± 2.55, 24.6 ± 5.09, 37.4 ± 1.68, 21.9 ± 1.76, 15.5 ± 1.04, and 9.9 ± 2.96 cm, respectively. 

The difference in plant length between treatments was highly significant, as revealed by one-way ANOVA (*p* < 0.001). Notably, the treatments varied widely in their impact on plant length, with specific treatments like 50% vermicompost and chemical fertilizers showing higher mean lengths, indicating their potential effectiveness; 10% FVWC and 10% BPRC gave similar results. Conversely, treatments such as 25% BPRC and 50% FRWC demonstrated lower mean lengths (Figure 1). 

Table 2 represents a multivariate analysis of variance (MANOVA) showing the effect of different factors on various plants’ growth parameters. MANOVA testing was conducted to compare the effects of different fertilizers on all plant consequences and their interactions. Concerning plant length, a highly significant difference was induced by MC (*p* < 0.001), FRWC (*p* < 0.001), VWC (*p* < 0.001), BPRC (*p* = 0.008), MFCWC (*p* < 0.001), and FVWC (*p* < 0.001). This result suggests that the type of fertilizer substantially impacted plant growth.

Moreover, the shoot length of *R. sativus* for different treatment groups from control, chemical fertilizers, vermicompost (10, 25, and 50%), VFWC (10, and 25%), BPRC (10, and 25%), MC (10, 25, and 50%), FRWC (10, 25, and 50%), MFCWC (10 and 25%), and VWC (10, and 25%), in terms of the mean ± standard deviation (SD) was 20.6 ± 0.91, 18.9 ± 0.82, 17.5 ± 1.63, 19.3 ± 0.56, 22.5 ± 2.07, 13.2 ± 0.83, 11.8 ± 0.69, 5.2 ± 0.29, 22.3 ± 0.75, 26.6 ± 1.25, 9.7 ± 2.36, 14.1 ± 1.39, 6.8 ± 0.75, 20.5 ± 1.42, 15.6 ± 3.18, 20.8 ± 2.45, 14.9 ± 0.37, 10.0 ± 0.68, and 6.7 ± 0.99 cm, respectively. 

The highest significant shoot lengths were recorded in 25% BPRC (26.6 ± 1.25 cm), followed by 10% BPRC (22.3 ± 0.75 cm), presented as mean ± SD. The difference in shoot length from different fertilizing treatments was highly significant (*p* < 0.001) (Figure 1). Furthermore, the root length of *R. sativus* for different treatment groups from control, chemical fertilizers, vermicompost (10, 25, and 50%), VFWC (10 and 25%), BPRC (10 and 25%), MC (10, 25, and 50%), FWC (10, 25, and 50%), MFCWC (10 and 25%), and VWC (10 and 25%), presented as the mean ± SD, was 15.5 ± 1.02, 16.1 ± 0.87, 12.3 ± 1.42, 11.8 ± 0.99, 17.8 ± 1.41, 11.6 ± 1.00, 8.7 ± 0.34, 2.6 ± 0.08, 9.1 ± 1.78, 8.3 ± 0.13, 8.2 ± 0.75, 10.0 ± 0.00, 4.4 ± 0.55, 9.1 ± 1.24, 9.1 ± 2.08, 14.0 ± 0.75, 8.1 ± 0.65, 9.0 ± 2.55, and 3.4 ± 0.70 cm, respectively (Figure 1).

**Figure 2:**
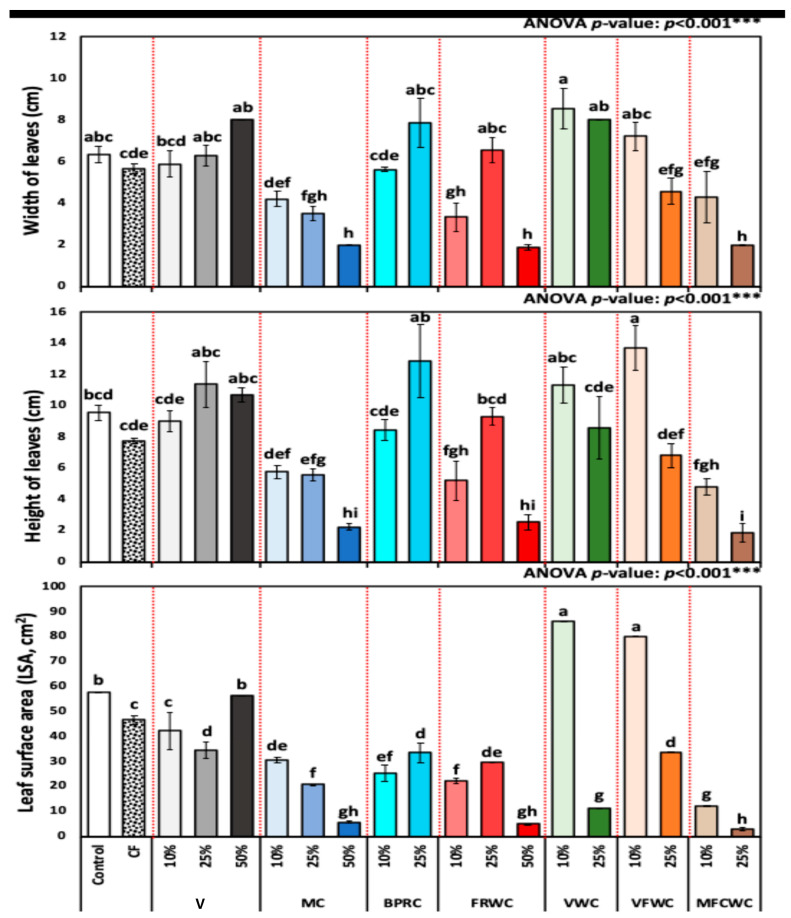
Leaf width (cm), height (cm), and surface area cm^2^ of *R. sativus* treated with different treatments, including chemical fertilizer (CF), vermicompost (V), mixed compost (MC), bread, pasta, and rice waste compost (BPRC), fruit waste compost (FRWC), vegetable waste compost (VWC), vegetable and fruit waste compost (VFWC), and meat, fish, and chicken waste compost (MFCWC). Bars followed by different letters are significantly different according to Tukey’s HSD at 0.05 level. ***: Highly significant at *p* < 0.001, as revealed by one-way ANOVA. The red dotted line differentiates between different treatments.

Figure 2 represents leaf width, height, and LSA in *R. sativus* treated with different treatments. Leaf widths of *R. sativus* plants at different treatments were 6.3 ± 0.38, 5.6 ± 0.24, 5.9 ± 0.61, 6.3 ± 0.49, 8.0 ± 0.00, 4.2 ± 0.35, 3.5 ± 0.34, 2.0 ± 0.00, 5.6 ± 0.13, 7.9 ± 1.20, 3.3 ± 0.69, 6.5 ± 0.59, 1.9 ± 0.12, 8.5 ± 0.99, 8.0 ± 0.00, 7.2 ± 0.66, 4.6 ± 0.63, 4.3 ± 1.22, and 2.0 ± 0.03 cm, respectively (Figure 2). 

Leaf heights of *R. sativus* plants at different treatment groups from control, chemical fertilizers, vermicompost (10, 25, and 50%), FVWC (10 and 25%), BPRC (10 and 25%), MC (10, 25, and 50%), FRWC (10, 25, and 50%), MFCWC (10 and 25%), and VWC (10 and 25%), in terms of the mean ± SD, were 9.5 ± 0.51, 7.7 ± 0.16, 9.0 ± 0.69, 11.4 ± 1.49, 10.7 ± 0.47, 5.8 ± 0.44, 5.6 ± 0.41, 2.2 ± 0.24, 8.5 ± 0.65, 12.9 ± 2.37, 5.2 ± 1.26, 9.3 ± 0.56, 2.5 ± 0.46, 11.3 ± 1.14, 8.6 ± 2.00, 13.7 ± 1.42, 6.8 ± 0.76, 4.8 ± 0.53, and 1.8 ± 0.60 cm, respectively (Figure 2). 

The LSA of *R. sativus* plants (mean ± SD) was 57.5 ± 0.00, 46.5 ± 1.84, 42.2 ± 7.51, 34.6 ± 3.34, 56.1 ± 0.00, 30.5 ± 1.00, 20.8 ± 0.01, 5.7 ± 0.25, 25.1 ± 3.26, 33.4 ± 3.82, 22.1 ± 1.21, 29.4 ± 0.00, 4.9 ± 0.02, 86.0 ± 0.00, 11.1 ± 0.00, 80.0 ± 0.00, 33.7 ± 0.00, 11.9 ± 0.23, and 2.9 ± 0.61 cm^2^, respectively (Figure 2). 

The results show considerable variation in leaf measurements among the different treatments. According to the MANOVA presented in Table 2, the leaf parameters showed significant differences in MC, VWC, FRWC, and MFCWC. The data shows significant variability in LSA among the treatments. For example, treatments like 10% VWC and 10% FVWC exhibited high mean leaf surface areas (86.0 ± 0.00 and 80.0 ± 0.00 cm^2^, respectively), suggesting their effectiveness in enhancing leaf growth. Conversely, treatments such as 25% MFCWC presented much lower mean LSA (2.93 ± 0.61 cm^2^) (Figure 2).

**Figure 3:**
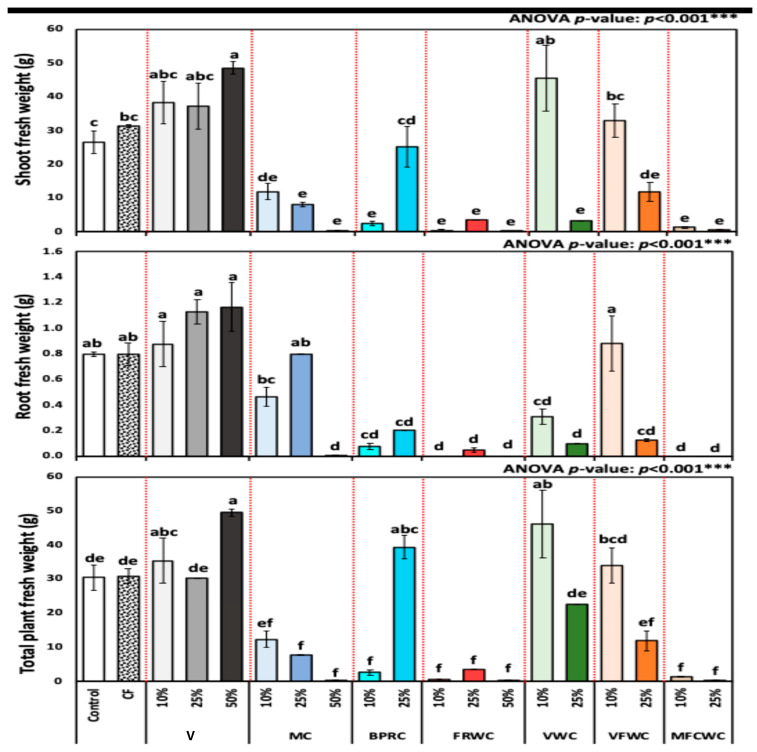
Shoot fresh weight (SFW) (g), root fresh weight (RFW) (g), and plant FW (PFW) (g) of *R. sativus* treated with different treatments, including chemical fertilizer (CF), vermicompost (V), mixed compost (MC), bread, pasta, and rice waste compost (BPRC), fruit waste compost (FRWC), vegetable waste compost (VWC), vegetable and fruit waste compost (VFWC), and meat, fish, and chicken waste compost (MFCWC). Bars followed by different letters are significantly different according to Tukey’s HSD at 0.05 level. ***: Highly significant at *p* < 0.001, as revealed by one-way ANOVA. The red dotted line differentiates between different treatments.

The plant biomass in terms of shoot fresh weight, root fresh weight, and plant fresh weight are presented in Figure 3. The shoot fresh weight of *R. sativus* from different treatment groups from control, chemical fertilizers, vermicompost (10, 25, and 50%), FVWC (10 and 25%), BPRC (10 and 25%), MC (10, 25, and 50%), FRWC (10, 25, and 50%), MFCWC (10 and 25%), and VWC (10 and 25%) (mean ± SD) was 26.5 ± 3.43, 31.4 ± 0.41, 38.2 ± 6.26, 37.3 ± 6.85, 48.6 ± 1.83, 11.9 ± 2.44, 8.0 ± 0.65, 0.4 ± 0.12, 2.5 ± 0.71, 25.1 ± 6.05, 0.5 ± 0.24, 3.5 ± 0.00, 0.3 ± 0.07, 45.5 ± 9.73, 3.2 ± 0.00, 33.0 ± 4.93, 11.9 ± 2.88, 1.3 ± 0.22, and 0.5 ± 0.24 g, respectively (Figure 3). The differences between all treatments were highly significant, as revealed by one-way ANOVA (*p* < 0.001).

Root fresh weight of *R. sativus* for different treatment groups from control, chemical fertilizers, vermicompost (10, 25, and 50%), FVWC (10 and 25%), BPRC (10 and 25%), MC (10, 25, and 50%), FRWC (10, 25, and 50%), MFCWC (10 and 25%), and VWC (10 and 25%) (mean ± SD) was 0.8 ± 0.02, 0.8 ± 0.09, 0.9 ± 0.18, 1.1 ± 0.10, 1.2 ± 0.19, 0.5 ± 0.07, 0.8 ± 0.00, 0.0 ± 0.00, 0.1 ± 0.03, 0.2 ± 0.00, 0.0 ± 0.00, 0.0 ± 0.02, 0.0 ± 0.00, 0.3 ± 0.06, 0.1 ± 0.00, 0.9 ± 0.21, 0.1 ± 0.01, 0.0 ± 0.00, and 0.0 ± 0.00 g, respectively (Figure 3). The differences between all fertilizers were highly significant, as revealed by one-way ANOVA (*p* < 0.001).

The plant fresh weight of *R. sativus* plants for different treatment groups (mean ± SD) from control, chemical fertilizers, vermicompost (10, 25, and 50%), FVWC (10 and 25%), BPRC (10 and 25%), MC (10, 25, and 50%), FRWC (10, 25, and 50%), MFCWC (10 and 25%), and VWC (10 and 25%) was 30.4 ± 3.62, 30.9 ± 2.11, 35.4 ± 6.57, 30.2 ± 0.00, 49.5 ± 1.12, 12.3 ± 2.46, 7.7 ± 0.06, 0.3 ± 0.07, 2.6 ± 0.71, 39.4 ± 3.40, 0.5 ± 0.24, 3.5 ± 0.00, 0.3 ± 0.07, 46.2 ± 9.93, 22.5 ± 0.00, 34.0 ± 5.22, 11.9 ± 2.95, 1.3 ± 0.22, and 0.2 ± 0.09 g, respectively (Figure 3). The differences between all fertilizers were highly significant, as revealed by one-way ANOVA (*p* < 0.001).

MANOVA, as delineated in Table 2, was performed to compare the effects of different independent factors of fertilizers (chemical, compost, vermicompost, FRWC, VWC, BPRC, MFCWC, and VFWC) and showed a highly significant effect of all included fertilizers except chemical fertilizer.

**Figure 4:**
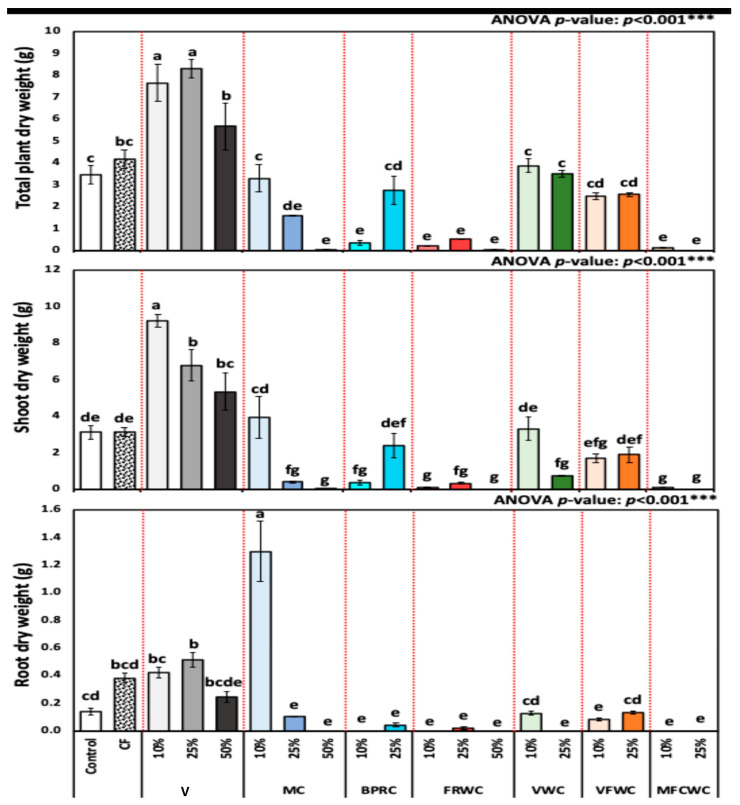
Shoot dry weight (SDW) (g), root dry weight (RDW) (g), and plant dry weight (PFW) (g) of *R. sativus* treated with different treatments, including chemical fertilizer (CF), vermicompost (V), mixed compost (MC), bread, pasta, and rice waste compost (BPRC), fruit waste compost (FRWC), vegetable waste compost (VWC), vegetable and fruit waste compost (VFWC), and meat, fish, and chicken waste compost (MFCWC). Bars followed by different letters are significantly different according to Tukey’s HSD at 0.05 level. ***: Highly significant at *p* < 0.001, as revealed by one-way ANOVA. The red dotted line differentiates between different treatments.

Figure 4 presents the mean and standard deviation for plant dry weight across different treatments. The dry weight of *R. sativus* for different treatment groups from control, chemical fertilizers, vermicompost (10, 25, and 50%), FVWC (10 and 25%), BPRC (10 and 25%), MC (10, 25, and 50%), FRWC (10, 25, and 50%), MFCWC (10 and 25%), and VWC (10 and 25%) control, chemical fertilizers, vermicompost (10, 25, and 50%), FVWC (10 and 25%), BPRC (10 and 25%), MC (10, 25, and 50%), FRWC (10, 25, and 50%), MFCWC (10 and 25%), and VWC (10 and 25%) (mean ± SD) was 2.2 ± 0.27, 2.6 ± 0.20, 5.8 ± 0.30, 5.2 ± 0.38, 3.7 ± 0.70, 2.7 ± 0.66, 0.7 ± 0.02, 0.0 ± 0.00, 0.3 ± 0.06, 1.6 ± 0.46, 0.1 ± 0.00, 0.3 ± 0.02, 0.0 ± 0.00, 2.4 ± 0.29, 1.4 ± 0.04, 1.4 ± 0.11, 1.5 ± 0.14, 0.1 ± 0.00, and 0.0 ± 0.00 g, respectively (Figure 4).

The results in Figure 4 demonstrated significant variations in dry weight among the treatments. For example, treatments like 10% vermicompost and 25% vermicompost showed higher mean dry weights (5.77 ± 0.91, and 5.21 ± 1.21 g, respectively), indicating their effectiveness in influencing plant dry weight (Figure 4). Regarding the treatment of fertilizers, 10% VWC gave the highest mean. In contrast, treatments such as 50% MC exhibited much lower mean dry weights (0.0126 ± 0.00 g).

**Figure 5:**
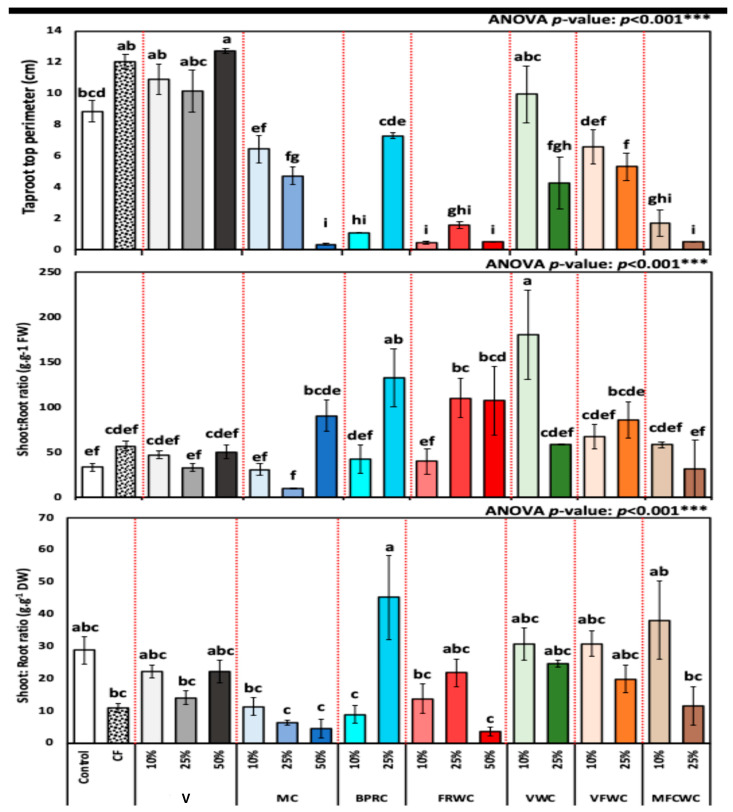
Taproot top perimeter (cm), biomass allocation, shoot:root ratio of fresh weight, and shoot:root ratio dry weight of *R. sativus* treated with different treatments, including chemical fertilizer (CF), vermicompost (V), mixed compost (MC), bread, pasta, and rice waste compost (BPRC), fruit waste compost (FRWC), vegetable waste compost (VWC), vegetable and fruit waste compost (VFWC), and meat, fish, and chicken waste compost (MFCWC). Bars followed by different letters are significantly different according to Tukey’s HSD at 0.05 level. ***: Highly significant at *p* < 0.001, as revealed by one-way ANOVA. The red dotted line differentiates between different treatments.

Figure 5 represents the variation in taproot top perimeter (cm) diameter, number of leaves, and mean leaves. The taproot top perimeter of *R. sativus* for different treatments (mean ± SD) control, chemical fertilizers, vermicompost (10, 25, and 50%), FVWC (10, and 25%), BPRC (10, and 25%), MC (10, 25, and 50%), FRWC (10, 25, and 50%), MFCWC (10, and 25%), and VWC (10, and 25%) was 8.9 ± 0.68, 12.0 ± 0.48, 10.9 ± 0.97, 10.2 ± 1.32, 12.7 ± 0.15, 6.4 ± 0.88, 4.7 ± 0.55, 0.4 ± 0.06, 1.1 ± 0.00, 7.3 ± 0.21, 0.5 ± 0.08, 1.6 ± 0.20, 0.5 ± 0.00, 10.0 ± 1.82, 4.3 ± 1.63, 6.6 ± 1.07, 5.3 ± 0.86, 1.7 ± 0.84, and 0.5 ± 0.00 cm, respectively (Figure 5). The data illustrate considerable variability in the taproot top perimeter.

The biomass allocation in terms of the shoot: root ratio was also assessed, and either calculated by FW or dry weight, both presented in Figure 5. The shoot: root ratio fresh weight of *R. sativus* for different treatment groups from control, chemical fertilizers, vermicompost (10, 25, and 50%), FVWC (10, and 25%), BPRC (10, and 25%), MC (10, 25, and 50%), FRWC (10, 25, and 50%), MFCWC (10, and 25%), and VWC (10, and 25%) (mean ± SD), control, chemical fertilizers, vermicompost (10, 25, and 50%), FVWC (10 and 25%), BPRC (10 and 25%), MC (10, 25, and 50%), FRWC (10, 25, and 50%), MFCWC (10 and 25%), and VWC (10 and 25%) was 33.4 ± 4.47, 56.9 ± 5.40, 47.4 ± 4.11, 33.1 ± 4.67, 50.6 ± 7.95, 30.9 ± 6.34, 10.1 ± 0.82, 90.9 ± 17.71, 42.7 ± 15.62, 132.9 ± 32.02, 39.9 ± 13.77, 110.2 ± 21.70, 107.4 ± 38.03, 181.1 ± 49.42, 58.6 ± 0.45, 67.7 ± 13.82, 86.1 ± 20.27, 58.4 ± 2.96, and 31.8 ± 31.83 g·g^−1^, respectively (Figure 5). 

The shoot: root ratio dry weight of *R. sativus* for different treatment groups from control, chemical fertilizers, vermicompost (10, 25, and 50%), FVWC (10, and 25%), BPRC (10, and 25%), MC (10, 25, and 50%), FRWC (10, 25, and 50%), MFCWC (10, and 25%), and VWC (10, and 25%) (mean ± SD) was 28.8 ± 4.22, 11.1 ± 1.37, 22.2 ± 2.02, 14.1 ± 2.11, 22.2 ± 3.59, 11.4 ± 2.85, 6.4 ± 0.80, 4.5 ± 2.99, 9.0 ± 2.71, 45.3 ± 13.06, 13.7 ± 4.59, 21.8 ± 4.20, 3.7 ± 1.41, 30.8 ± 5.16, 24.6 ± 1.03, 30.9 ± 3.94, 19.8 ± 4.27, 38.2 ± 12.23, and 11.5 ± 5.92 g·g^−1^, respectively (Figure 5). The most significant biomass allocated toward shoots in terms of fresh wight was recorded in 10% VWC and 25% BPRC, which were 181.13, and 132.89 g·g^−1^, respectively (Figure 5). However, the lowest was recorded in 25% MC and 10% MC, at 10.07 and 30.89 g·g^−1^, which proves the improvement in biomass towards the root system, which is more important in *R. sativus* (Figure 5).

This proves an improved allocation towards shoot biomass by 10% VWC and 25% BPRC. However, based on dry weight, the highest significant biomass allocations were in 25% BPRC and 10% MFCWC, of 45.29, and 38.20 g·g^−1^, respectively (Figure 5). The lowest shoot: root ratio based on dry weight was recorded in 50% FRWC, 50% MC, and 25%, MC with an average shoot: root ratio of 3.67, 3.83, 4.50, and 6.44 g·g^−1^ dry weight, respectively (Figure 5).

Table 3 shows the effect of different fertilizers on soil properties. pH is an essential factor affecting microbial growth and reproduction; adding fertilizers to the soil modified pH availability and EC for plants. The pH ranged from 5.75 to 8.52 using 10% FRWC and 50% MFCWC, respectively (Table 3). 

Table 4 presents the total bacterial population with the unit colony-forming unit (CFU). The highest numbers were for 25% MFCWC, 10% vermicompost, and 25% vermicompost.

## 4. Discussion

The current study provides useful insights into the possible use of various kinds of food waste as fertilizers in organic farming. The present findings contribute to an emerging body of research that promotes sustainable agriculture practices and offers practical answers for waste management and environmental conservation.

The findings obtained from the present investigation are consistent with similar studies that have been conducted on vermicompost and composts made from food waste [21,22,23,27]. Transforming food waste into valuable soil amendments, such as compost, vermicompost, biofertilizer, biochar, and engineered biochar, is an optimal strategy for recovering and reusing nutrient-rich organic waste [27,42]. These amendments have the potential to enhance soil fertility and crop yield by serving as direct sources of essential nutrients (such as carbon, nitrogen, phosphorus, potassium, calcium, iron, and zinc), and/or by improving nutrient availability through alterations in soil porosity, water retention, surface interactions, soil pH, and cation exchange capacity [42,43].

Food wastes are typically abundant in nitrogen and can be utilized in soils that lack nutrients. O‘Connor et al., 2022 [44] demonstrated that dehydrated vegetables exhibited elevated levels of total nitrogen and plant-available nitrogen. Consequently, they can serve as a fertilizer to enhance crop growth, among other applications [14,44,45,46]. Another study investigated the effectiveness of recycling kitchen waste as an organic nitrogen fertilizer for sustainable agriculture in cool and warm seasons [19]. The results indicated that kitchen waste outperformed mineral fertilizer as a fertilizer, but this effect was only observed during the cool season. Furthermore, it resulted in a 20–40% increase in plant yields for nitrogen. Introducing kitchen waste into the soil yielded superior soil characteristics compared to mineral fertilizer [19].

A separate study investigated the efficacy of an organic liquid fertilizer derived from recycled food wastes when applied to lettuce, cucumber, and cherry tomatoes in hydroponic systems [21]. It compared its performance to that of a commercially available liquid fertilizer [21]. The nitrogen and phosphorus concentrations in these plant structural components are comparable between recycled food fertilizer and commercial liquid fertilizer [21]. These findings indicated that recycled food fertilizer could be a viable substitute for commercial liquid fertilizer in hydroponic systems used for lettuce and cucumber [21]. In addition to using food waste as a fertilizer, other researchers use recycling techniques to produce both chicken feed and liquid fertilizer (CFLF). The liquid extract from the CFLF process exhibited elevated nutrient concentrations comparable to those found in feed solutions utilized in hydroponic systems [47]. Consequently, the liquid extract derived from CFLF possesses the potential to serve as a substitute for the commercially available liquid fertilizer commonly employed in hydroponic systems [47].

Vermicompost and compost are recognized for their capacity to improve plant growth and combat abiotic antistress. Additionally, they enhance the level of potassium in soil and experimental plants [13]. The morphophysiological traits of tomatoes, including leaf length, plant height, leaf width, leaf count, flowering time, number of primary branches per plant, stem diameter, fruit diameter, fruit count per plant, and petiole length, were improved with the vermicompost compared to chemical fertilizer [48]. An innovative approach involves utilizing dairy waste as a nutritional source for wheat plants; dairy waste can be used as an organic fertilizer, which results in significantly enhanced quality [49]. The extracted organic fertilizer demonstrated a significant advantage over mineral fertilization by effectively meeting the nutrient needs of wheat [49].

In line with our findings, it has also been observed that different compost and vermicompost treatments have an impact. Previous research demonstrated that a fertilizer derived from food waste had the following properties: EC of 6.36 mS/cm and a pH of 6.5, and the food waste also positively impacted plant nutrient levels; for example, it affected the plant height, number of leaves, length, and width of leaves, and the bioactivity of pineapple [13,50]. Another study found that it also affects the length of the shoot, length of internode, number of leaves, and number of branches in *Capsicum annum* [51]. 

Additional composting is an effective strategy for converting agricultural and urban waste into forms that benefit crops. A study investigating the impact of using recycled compost on soil food webs, nutrient cycling, and tree growth in a young almond orchard showed that both dairy manure compost and food waste compost resulted in increased pools, soil, and ammonium levels [52]. Both composts had a noticeable impact on bacterial communities following application, particularly on groups capable of breaking down carbon [52]. They resulted in increased nematodes that feed on bacteria at different intervals. Distinct associations were found between nematode and bacterial groups in compost treatments, which were absent in the control group [52]. The application of food waste caused an increased trunk perimeter compared to the control group. Additionally, the compost had a higher relative abundance of nematodes that feed on the tips of herbivorous roots [52]. These findings indicated that using food waste supports the process of biological nitrogen cycling and can potentially promote tree growth, particularly in the initial year following application [52].

A study carried out by Loera-Muro et al. (2021) [53] examined the morphology, precisely root length, and microbial traits during the initial growth stage of mint and rosemary plants. The research aimed to cultivate lettuce using vermicompost and thermophilic compost. According to Schröder et al. (2021) [54], the lettuce crop yielded the highest amount of phosphorus, potassium, calcium, and magnesium in vermicompost made from coir-based vermicompost [54]. 

The presence of small sample sizes in our treatment groups may compromise the generalizability of the findings. Although attempts were made to regulate extraneous variables, fluctuations in environmental conditions may have influenced the outcomes.

## 5. Conclusions

The current study revealed that the use of organic food waste fertilizers has a positive impact on both sustainable agriculture and waste management. The present investigation indicated that the application of vermicompost, FOWC, and other organic amendments positively impacted the growth of shoots and roots, as well as the overall biomass and LSA, of red radish. Vermicompost enhanced plant metrics, particularly when applied at a concentration of 50%, indicating its potential as a superior organic fertilizer. Due to the ineffectiveness of higher compost concentrations, a balanced fertilizer is necessary. Utilizing food waste as agricultural inputs enhances soil quality, diminishes the need for chemical fertilizers, and fosters environmental stewardship, thereby contributing to global sustainability objectives.

Expanding upon its basic findings, the current study suggests other areas for future research that should be explored:

1. Assessment of the long-term agricultural effects by examining soil health and composition using fertilizers derived from food waste across multiple growth cycles. This would demonstrate the long-lasting effectiveness of organic amendments.
2. Examination of economic feasibility by comparing the cost-effectiveness of food waste and chemical fertilizers. The analysis should consider the costs of production, the efficiency of application, and the increases in yield to ascertain the economic accessibility for farmers.
3. Comprehensive crop testing by conducting tests on a wider range of crops can help determine the suitability of using food waste as fertilizer in various agricultural settings. This would aid in assessing crop requirements and optimizing fertilizer compositions.
4. Optimizing composting techniques and formulations is necessary to maximize the nutrient content and promote soil health. This encompasses examining both food waste and composting. The impact of these fertilizers on soil and plant water retention could have significant implications for water conservation strategies in arid agricultural regions.
5. Conducting an environmental impact assessment to assess the greenhouse gas emissions of fertilizers derived from food waste. This will assess the ecological footprint of these fertilizers compared to traditional fertilizers. It is imperative to establish safety regulations for food waste fertilizers to ensure their safety, efficacy, and environmental friendliness.
6. Implementation of educational programs to educate farmers and the public about the advantages and methods of using food waste as fertilizer, thereby accelerating its acceptance and implementation.

Implementing crop rotation, organic farming, and precision agriculture techniques can enhance the productivity and sustainability of farming by using food waste as fertilizer. These suggestions will aid future research in verifying and enhancing the utilization of food waste as a valuable resource, thereby fostering sustainable agriculture on a global scale. These endeavors employ waste recycling methods and environmental conservation practices to enhance agricultural output and establish a sustainable economic system.

## Figures and Tables

**Table 1:** Experimental layout under greenhouse conditions.

	Treatments	VWC	FRWC	VFWC	MFCWC	BPRC	MC	V	CF	C
Percentage										
Experiment layout	10%	*8 (2)**	8 (2)	8 (2)	8 (2)	8 (2)	8 (2)	8 (2)	8 (2)	8 (2)
	25%	8 (2)	8 (2)	8 (2)	8 (2)	8 (2)	8 (2)	8 (2)	8 (2)	8 (2)
	50%	8 (2)	8 (2)	8 (2)	8 (2)	8 (2)	8 (2)	8 (2)	8 (2)	8 (2)

The experiment was conducted using a split–split plot design, where food waste compost treatments (FOWC), vermicompost, chemical fertilizer, and control treatments were randomly assigned to sub-plots. Each experimental sub-plot comprised eight rows of plots, with three replicates arranged randomly. Two seeds were sown in each pot. VWC, vegetable waste compost; FRWC, fruit waste compost; VFWC, vegetable, and fruit waste compost; MFCWC, meat, fish, and chicken waste compost; BPRC, bread, pasta, and rice waste compost; MC, mixed green and brown materials compost; V, Vermicompost; CF, chemical fertilizer; C, control. 10% = 10% of the treatments and 90% from seed starter potting mix; 25% = 25% of the treatments and 75% from seed starter potting mix, and 50% = 50% of the treatments and 50% from seed starter potting mix. *8: eight rows, (2)** two seeds in each pot.

**Table 2:** Multivariate analysis of variance (MANOVA) presents the effect of different factors on various plants’ growth parameters.

Plant Parameter *	Corrected Model		CF		MC		V		FRWC		VFWC		BPRC		MFCWC		VWC	
	F	*p*-Value	F	*p*-Value	F	*p*-Value	F	*p*-Value	F	*p*-Value	F	*p*-Value	F	*p*-Value	F	*p*-Value	F	*p*-Value
Plant length (cm)	20.9	<0.001 ***	0.7	0.396 ns	44.0	<0.001 ***	2.4	0.065 ns	11.4	<0.001 ***	8.6	<0.001 ***	4.9	0.008 **	32.5	<0.001 ***	18.8	<0.001 ***
Root length (cm)	11.8	<0.001 ***	0.5	0.469 ns	25.8	<0.001 ***	4.3	0.006 **	6.1	<0.001 ***	3.1	0.048 *	13.9	<0.001 ***	15.6	<0.001 ***	11.2	<0.001 ***
Shoot length (cm)	12.1	<0.001 ***	1.2	0.275 ns	24.1	<0.001 ***	1.8	0.148 ns	8.9	<0.001 ***	7.5	<0.001 ***	6.1	0.003 **	16.7	<0.001 ***	6.7	0.002 **
Taproot top perimeter (cm)	16.0	<0.001 ***	13.6	<0.001 ***	15.9	<0.001 ***	3.3	0.022 *	12.6	<0.001 ***	9.3	<0.001 ***	9.3	<0.001 ***	17.0	<0.001 ***	12.6	<0.001 ***
Number of leaves	4.4	<0.001 ***	1.9	0.166 ns	7.7	<0.001 ***	1.2	0.324 ns	2.4	0.065 ns	7.5	<0.001 ***	1.8	0.161 ns	4.3	0.014 *	3.2	0.041 *
Width of leaves (cm)	10.0	<0.001 ***	1.3	0.263 ns	13.1	<0.001 ***	2.1	0.103 ns	14.3	<0.001 ***	8.1	<0.001 ***	4.6	0.012 *	7.1	0.001 ***	7.5	<0.001 ***
Height of leaves (cm)	10.5	<0.001 ***	3.9	0.050 *	12.7	<0.001 ***	0.5	0.683 ns	5.8	<0.001 ***	13.7	<0.001 ***	7.5	<0.001 ***	10.1	<0.001 ***	6.9	0.001 ***
Total fresh weight (g)	13.9	<0.001 ***	0.3	0.601 ns	14.4	<0.001 ***	5.0	0.002 **	14.1	<0.001 ***	17.5	<0.001 ***	9.8	<0.001 ***	13.5	<0.001 ***	3.3	0.040 *
Shoot fresh weight (g)	14.2	<0.001 ***	3.2	0.076 ns	9.9	<0.001 ***	7.4	<0.001 ***	5.9	<0.001 ***	27.9	<0.001 ***	5.1	0.007 **	10.1	<0.001 ***	9.1	<0.001 ***
Root fresh weight (g)	13.3	<0.001 ***	0.0	0.913 ns	13.0	<0.001 ***	2.7	0.044 *	2.4	0.072 ns	5.0	0.008 **	15.0	<0.001 ***	15.5	<0.001 ***	22.6	<0.001 ***
Total plant dry weight (g)	16.8	<0.001 ***	3.5	0.064 ns	11.0	<0.001 ***	26.2	<0.001 ***	11.3	<0.001 ***	6.4	0.002 **	5.2	0.006 **	12.0	<0.001 ***	3.1	0.047 *
Shoot dry weight (g)	13.4	<0.001 ***	0.1	0.809 ns	14.0	<0.001 ***	22.1	<0.001 ***	4.1	0.007 **	3.1	0.046 *	3.1	0.048 *	7.3	<0.001 ***	7.0	0.001 ***
Root dry weight (g)	8.8	<0.001 ***	9.6	0.002 **	34.0	<0.001 ***	3.4	0.020 *	0.3	0.828 ns	0.1	0.932 ns	0.6	0.576 ns	0.8	0.457 ns	0.9	0.426 ns
Total plant length	21.1	<0.001 ***	1.0	0.323 ns	44.8	<0.001 ***	3.1	0.028 *	14.1	<0.001 ***	9.3	<0.001 ***	4.0	0.021 *	33.1	<0.001 ***	17.6	<0.001 ***
Leaf surface area (LSA, cm^2^)	78.8	<0.001 ***	34.4	<0.001 ***	109.7	<0.001 ***	12.8	<0.001 ***	43.0	<0.001 ***	215.6	<0.001 ***	26.0	<0.001 ***	102.4	<0.001 ***	43.1	<0.001 ***

* Chemical fertilizer (CF), mixed compost (MC), vermicompost (V), fruit waste compost (FRWC), vegetable and fruit waste compost (VFWC), bread, pasta, and rice waste compost (BPRC), meat, fish, and chicken waste compost (MFCWC), and vegetable waste compost (VWC). **: highly significant at *p* < 0.010; ***: highly significant at *p* < 0.001; ns: non-significant at *p* > 0.05.

**Table 3:** Effect of different treatments on soil pH and soil electrical conductivity (EC).

Treatments	pH	EC mS/cm
Vegetable waste compost VWC (10%)	6.84	3.81
Vegetable waste compost VWC (25%)	7.07	5.36
Vegetable waste compost VWC (50%)	7.27	6.07
Fruit waste compost FRWC (10%)	5.75	2.94
Fruit waste compost FRWC (25%)	6.66	3.73
Fruit waste compost FRWC (50%)	6.97	4.47
Vegetable and fruit waste compost VFWC (10%)	6.77	7.17
Vegetable and fruit waste compost VFWC (25%)	7.52	5.11
Vegetable and fruit waste compost VFWC (50%)	8.04	3.28
Meat, fish, and chicken waste compost (MFCWC 10%)	6.09	5.53
Meat, fish, and chicken waste compost (MFCWC 25%)	7.65	5.39
Meat, fish, and chicken waste compost (MFCWC 50%)	8.52	6.25
Bread, pasta, and rice waste compost (BPRC 10%)	6.5	6.25
Bread, pasta, and rice waste compost (BPRC 25%)	6.85	5.24
Bread, pasta, and rice waste compost (BPRC 50%)	7.31	3.67
Mixed compost (MC 10%)	6.65	2.71
Mixed compost (MC 25%)	6.52	4.57
Mixed compost (MC 50%)	6.44	8.74
Vermicompost (V 10%)	6.47	2.04
Vermicompost (V 25%)	6.73	1.2
Vermicompost (V 50%)	6.83	2.79
Chemical Fertilizer (CF)	6.7	1.12
Control (C)	6.4	0.53

Vegetable waste compost (VWC), fruit waste compost (FRWC), vegetable and fruit waste compost (VFWC), meat, fish, and chicken waste compost (MFCWC), bread, pasta, and rice waste compost (BPRC), mixed compost (MC), vermicompost (V), chemical fertilizer (CF), and control (C).

**Table 4:** Effect of different treatments on total bacterial population in soils.

Treatments	Total Bacterial Population Colony-Forming Units (×10^5^)
Vegetable waste compost VWC (10%)	20
Vegetable waste compost VWC (25%)	75
Vegetable waste compost VWC (50%)	144
Fruit waste compost FRWC (10%)	22
Fruit waste compost FRWC (25%)	61
Fruit waste compost FRWC (50%)	27
Vegetable and fruit waste compost VFWC (10%)	49
Vegetable and fruit waste compost VFWC (25%)	42
Vegetable and fruit waste compost VFWC (50%)	25
Meat, fish, and chicken waste compost (MFCWC 10%)	15
Meat, fish, and chicken waste compost (MFCWC 25%)	211
Meat, fish, and chicken waste compost (MFCWC 50%)	16
Bread, pasta, and rice waste compost (BPRC 10%)	0
Bread, pasta, and rice waste compost (BPRC 25%)	18
Bread, pasta, and rice waste compost (BPRC 50%)	136
Mixed compost (MC 10%)	113
Mixed compost (MC 25%)	3
Mixed compost (MC 50%)	20
Vermicompost (V 10%)	208
Vermicompost (V 25%)	162
Vermicompost (V 50%)	140
Chemical Fertilizer (CF)	6
Control (C)	1

Vegetable waste compost (VWC), fruit waste compost (FRWC), vegetable and fruit waste compost (VFWC), meat, fish, and chicken waste compost (MFCWC), bread, pasta, and rice waste compost (BPRC), mixed compost (MC), vermicompost (V), chemical fertilizer (CF), and control (C).

## Data Availability

The original contributions presented in the study are included in the article, further inquiries can be directed to the corresponding author.
